# Metabarcoding of pathogenic parasites based on copro-DNA analysis of wild animals in South Korea

**DOI:** 10.1016/j.heliyon.2024.e30059

**Published:** 2024-04-25

**Authors:** Jun Ho Choi, Soo Lim Kim, Dong Kyun Yoo, Myung-hee Yi, Singeun Oh, Myungjun Kim, Sohyeon Yun, Tai-Soon Yong, Seongjun Choe, Jong Koo Lee, Ju Yeong Kim

**Affiliations:** a Department of Tropical Medicine, Institute of Tropical Medicine, and Arthropods of Medical Importance Resource Bank, Yonsei University College of Medicine, Yonsei-ro 50-1, Seodaemun-gu, Seoul, 03722, Republic of Korea; b Division of Life Science, Incheon National University, 119 Academy-ro, Yeonsu-gu, Incheon, 22012, Republic of Korea; c Department of Parasitology, School of Medicine, Chungbuk National University, Cheongju, 28644, Republic of Korea

**Keywords:** Parasitic infection, Wild animals, Molecular identification, 18S rRNA gene, South Korea

## Abstract

Four species of dominant wild animals, namely, *Prionailurus bengalensis euptilurus*, *Nyctereutes procyonoides koreensis*, *Hydropotes inermis argyropus*, and *Sus scrofa coreanus*, are hosts of potential infectious agents, including helminths and protozoa. Therefore, it is necessary to analyze the infectious agents present in these wild animals to monitor and control the spread of pathogens. In the present study, fecal samples from 51 wild animals were collected from the mountains of Yangpyeong, Hoengseong, and Cheongyang in South Korea and metabarcoding of the V9 region of the 18S rRNA gene was performed to identify various parasite species that infect these wild animals. Genes from nematodes, such as *Metastrongylus* sp., *Strongyloides* spp., *Ancylostoma* sp., and *Toxocara* sp., were detected in the fecal samples from wild animals. In addition, platyhelminthes, including *Spirometra* sp., Echinostomatidae gen. sp., *Alaria* sp., *Neodiplostomum* sp.*,* and *Clonorchis* sp., and protozoa, including *Entamoeba* sp., *Blastocystis* sp., *Isospora* sp., *Tritrichomonas* sp., *Pentatrichomonas* sp.*,* and *Cryptosporidium* sp., were detected. In the present study, various parasites infecting wild animals were successfully identified using metabarcoding. Our technique may play a crucial role in monitoring parasites within wild animals, especially those causing zoonoses.

## Highlights

• Feces from 51 wild animals of South Korea were analyzed by 18S rRNA gene amplicon sequencing.
• Species of helminths and protozoa were detected in the fecal samples.
• Better management of transmission of infectious agents from wild animals could be achieved by implementing metabarcoding.

## Graphical abstract

**Figure:**
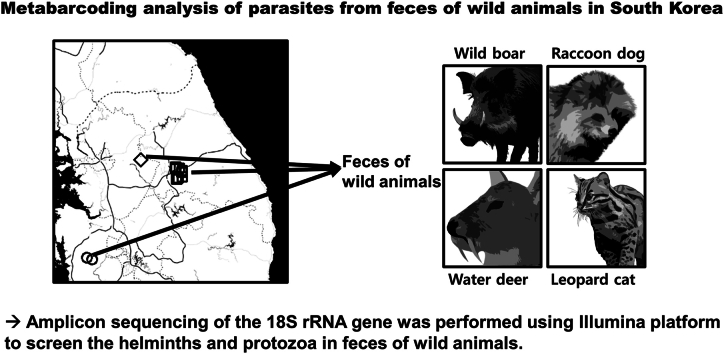

ASV: Amplicon sequence variants
GPS: Global positioning system
OTU: Operational taxonomic unit
PCR: Polymerase chain reaction

## 1 Introduction

Wild animals are important potential hosts for transporting and transmitting infectious pathogens, such as helminths and protozoa [1]. They can spread the infectious diseases caused by these pathogens to humans, livestock, and other wild animal populations, even over great distances [2,3]. Wild animals are also known to carry zoonotic diseases [4,5]. Such diseases can spread to humans through contact with infected wild animals, their feces, or other animals that have been exposed to them. Uncontrolled migration of wild animals into urban areas also creates an additional threat, including the risk of contamination of water, food, and soil with parasite eggs/oocysts [6].

The repercussions of zoonotic diseases span a broad spectrum, affecting livestock, the economy, and human health. Roughly 75 % of emerging infectious diseases are zoonotic in nature [7]. Such diseases affect livestock, which may lead to decreased productivity, reduced fertility, and diminished meat, milk, and wool outputs, as well as diminished quality or loss of human life [8].

Four species of wild animals, *Prionailurus bengalensis euptilurus*, *Nyctereutes procyonoides koreensis*, *Hydropotes inermis argyropus*, and *Sus scrofa coreanus*, which are widespread in South Korean wildlands, have the potential to carry and transmit various infectious pathogens that can harm humans and other animals. Up to 700,000 individuals of *H. inermis argyropus,* the Korean water deer, inhabit various regions across South Korea [9], and can carry *Giardia duodenalis* [10] and *Cryptosporidium* spp. [11]. *P. bengalensis euptilurus*, also known as the leopard cat, is a small wild cat that is native to Korea; it can carry *Toxoplasma gondii* [12], a protozoan that causes human toxoplasmosis. *N. procyonoides koreensis*, also known as the Korean raccoon dog, is a host of the nematode *Trichinella spiralis* [13], which causes trichinosis in humans. It can also carry *Cryptosporidium parvum* [14], a protozoan that causes cryptosporidiosis in humans. Lastly, *S. scrofa coreanus*, also known as the wild boar, is a prime host for the nematode *Trichinella spiralis* [15], which can cause human trichinosis; wild boars can also carry *T. gondii* [16].

Using metabarcoding to analyze parasites in feces offers several advantages over traditional methods, such as microscopic inspection and conventional PCR. Firstly, by the nature of the metabarcoding approach, a single run can detect all types of parasites present in a sample, including certain protozoans that might be missed by microscopic examination. Additionally, even parasites that are hard to identify can be detected using a universal primer. Furthermore, with the recent reduction in the cost of next-generation sequencing (NGS), metabarcoding has become more economical in terms of money, labor, and time.

The aim of the present study was to identify helminthic and protozoal parasite species infecting four common wild animal species in South Korea by metabarcoding the 18S rRNA gene. The objective was to determine the prevalence of infections caused by various protozoal and helminthic parasites in wild animals within South Korea. The study was conducted using fecal samples collected from 51 wild animals among four species (*P. bengalensis euptilurus*, *N. procyonoides koreensis*, *H. inermis argyropus*, and *S. scrofa coreanus*) from the mountains of Yangpyeong, Hoengseong, and Cheongyang.

## 2 Methods

### 2.1 Sampling location

**Fig. 1:**
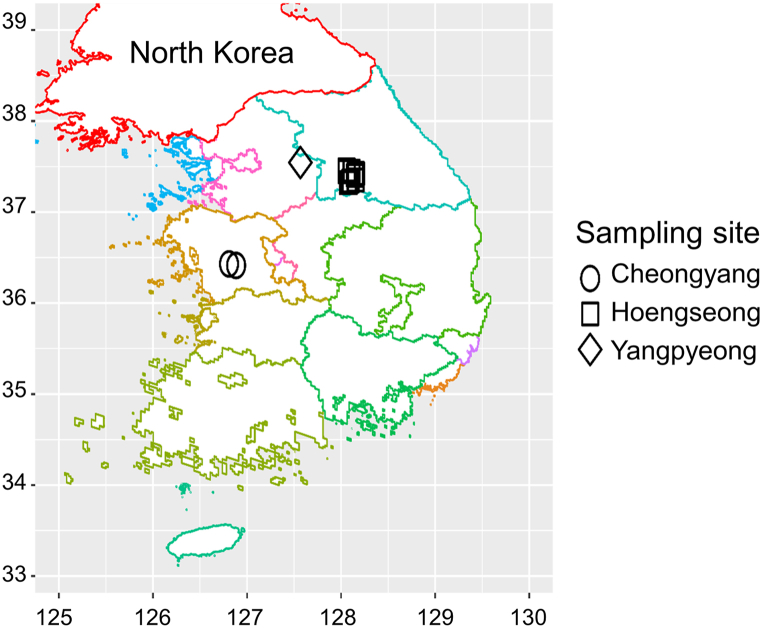
Research area and sampling sites where fecal samples of wild animals were collected. Provinces are differentiated by colors, while sampling sites are represented by distinct shapes: circles for Cheongyang, squares for Hoengseong, and diamonds for Yangpyeong. Detailed information, such as the collection date and global positioning system (GPS) coordinates, are presented in Supplementary Table S1. (For interpretation of the references to color in this figure legend, the reader is referred to the Web version of this article.)

Fecal samples from 51 wild animals, including five wild boars, 28 water deer, 13 raccoon dogs, and five leopard cats, were collected from the mountains in Hoengseong, Cheongyang, and Yangpyeong in South Korea (Fig. 1). Supplementary Table S1 provides information on the collection date and global positioning system (GPS) coordinates of the wild animals used in this study.

### 2.2 Collection of fecal samples and DNA extraction

After examining the morphologies of the fecal samples, each sample was stored in an individual container. The stool samples were delivered to the laboratory within a day of collection. DNA was extracted from all of the fecal samples using the FastDNA SPIN kit for soil (MP Biomedicals, Carlsbad, CA, USA), and the samples were stored at −80 °C until further use.

### 2.3 Illumina sequencing

For eukaryotic studies, the V9 region of 18S rRNA gene was amplified by polymerase chain reaction (PCR) using the primers, 1391f (5′-TCGTCGGCAGGTCAGGTATGTATAGGTAGOGAGGTACACACCCCGTCTACHC-3′) and EukBr (5′-GTCTCGTGGGGGGCGTAGGTAGTATAGACAGTGACHTTCAGGTCTCTAC-3′) [17]. To confirm the animal fecal source, the 12S rRNA genes were identified by PCR using primers, L1085 (5′-TCGTCGGCAGCGTCAGATGTGTATAAGAGACAGCCCAAACTGGGATTAGATACCC-3′) and H1259 (5′-GTCTCGTGGGCTCGGAGATGTGTATAAGAGACAGGTTTGCTGAAGATGGCGGTA-3′) [18]. The amplicon library was deep-sequenced using the Illumina iSeq™ 100 sequencing system (Illumina Inc., San Diego, CA, USA) according to the method described in our previous study [19].

### 2.4 Bioinformatics

For bioinformatics analysis, the standard DADA2 denoising pipeline [20] from Qiime2 version 2022.2 [21] was used for demultiplexing, forward and reverse paired-end read merges, quality filtering, and chimeric sequence removal to generate feature tables of amplicon sequence variants (ASVs). For the taxonomic classification of ASV sequences [22], all of the sequences included in the NCBI nucleotide database (https://www.ncbi.nlm.nih.gov/nuccore/) were used to build a database of vertebrates and parasites. To do this, an advanced search for gene names, “12S rRNA” or “18S rRNA” [18] was performed and sequences from the NCBI database were obtained. Clustered sequences with 95 % identity were compared with 12S rRNA and 18S rRNA sequences from the database to create a classification table. Matches were determined for the vertebrate with the highest identity, lowest e-value, and best result per read. 12S rRNA was only analyzed for ASVs in animal-accurate species identification, and 18S rRNA was only analyzed for ASVs in parasites. Sequences of arthropods, chordates, and fungi were removed. In addition, sequences with an operational taxonomic unit (OTU) number of 10 or less were excluded as thresholds.

## 3 Results

Using 18S rRNA amplicon sequencing, we obtained 67,242 reads per sample (Supplementary Table S2). Accurate identification of the species from which the fecal samples were obtained was confirmed by 12S rRNA amplicon sequencing (Supplementary Table S3). The morphologies of the animal feces were matched with the results from the 12S rRNA gene analysis in all samples.

**Table 1:** Parasites detected in the feces of wild boars (n = 5) by metabarcoding the V9 region of the 18S rRNA gene.

	Parasites	Number of positive fecal samples (%)	Average reads	Location	Animal ID
Helminths	*Metastrongylus* sp.	3 (60.00 %)	823.40	Hoengseong	S001, S004, S005
	*Strongyloides* sp. 1	3 (60.00 %)	71.20	Hoengseong	S001, S003, S004
Protozoa	*Entamoeba* sp.	4 (80.00 %)	55.00	Hoengseong	S001, S002, S003, S005
	*Blastocystis* sp.	1 (20.00 %)	295.20	Hoengseong	S001
	*Pentatrichomonas* sp.	1 (20.00 %)	10.20	Hoengseong	S004

**Table 2:** Parasites detected in the feces of raccoon dogs (n = 13) by metabarcoding of the V9 region of the 18S rRNA gene.

	Parasites	Number of positive fecal samples (%)	Average reads	Location	Animal ID
Helminths	*Ancylostoma* sp.	6 (46.26 %)	259.54	Hoengseong	S006, S007, S011, S012, S015, S016
	*Strongyloides* sp. 2	5 (40.99 %)	22.46	Hoengseong	S006, S007, S011, S012, S016
	*Toxocara* sp.	5 (40.34 %)	34.62	Hoengseong	S006, S009, S010, S011, S016
	*Panagrellus* sp.	1 (7.69 %)	2.08	Cheongyang	S017
	Echinostomatidae gen. sp.	2 (15.38 %)	20.00	Hoengseong, Cheongyang	S006, S018
	*Alaria* sp.	2 (15.38 %)	34.62	Hoengseong	S006, S012

In wild boars, *Metastrongylus* sp. and *Strongyloides* sp. 1 were detected with a prevalence of 60 % (Table 1). Infections with the protozoan *Entamoeba* sp. (80 %), *Blastocystis* sp. (20 %), and *Pentatrichomonas* sp. (20 %) were also detected in wild boars (Table 1).

In raccoon dogs, *Ancylostoma* sp. (46.26 %), *Strongyloides* sp. 2 (40.99 %), and *Toxocara* sp. (40.34 %) were detected (Table 2). In addition, two types of trematodes, Echinostomatidae gen. sp. (15.38 %) and *Alaria* sp. (15.38 %) were detected in raccoon dogs (Table 2).

In the present study, *Strongyloides* sp. 2 (40 %) and *Ancylostoma* sp. (20 %) were detected in leopard cats (Table 3). In addition, *Spirometra* sp. (40 %) and three types of trematodes, Echinostomatidae gen. sp. (20 %), *Neodiplostomum* sp. (20 %), and *Clonorchis* sp. (20 %) were detected in leopard cats (Table 3). The protozoans *Isospora* sp. (20 %), *Tritrichomonas* sp. (20 %), and *Cryptosporidium* sp. (20 %) were also detected in the fecal samples of leopard cats (Table 3).

**Table 3:** Parasites detected in the feces of leopard cats (n = 5) by metabarcoding of the V9 region of the 18S rRNA gene.

	Parasites	Number of positive fecal samples (%)	Average reads	Location	Animal ID
Helminths	*Strongyloides* sp. 2	2 (40.00 %)	631.60	Hoengseong, Yangpyeong	S019, S023
	*Ancylostoma* sp.	1 (20.00 %)	5.00	Cheongyang	S022
	*Spirometra* sp.	2 (40.00 %)	485.40	Cheongyang, Yangpyeong	S022, S023
	Echinostomatidae gen. sp.	1 (20.00 %)	53.20	Yangpyeong	S023
	*Neodiplostomum* sp.	1 (20.00 %)	10.20	Hoengseong	S019
	*Clonorchis* sp.	1 (20.00 %)	7.60	Yangpyeong	S023
Protozoa	*Isospora* sp.	1 (20.00 %)	114.80	Hoengseong	S019
	*Tritrichomonas* sp.	1 (20.00 %)	11.60	Yangpyeong	S023
	*Cryptosporidium* sp.	1 (20.00 %)	4.40	Yangpyeong	S023

In water deer, *Strongyloides* sp. 1 was detected with a prevalence of 8.71 % (Table 4). *Oscheius* sp. was only detected in one sample (3.57 %). The protozoans *Entamoeba* sp., (57.14 %), *Sappinia* sp. (3.57 %), and *Eimeria* sp. (3.57 %) were detected in water deer (Table 4).

**Table 4:** Parasites detected in the feces of water deer (n = 28) by metabarcoding of the V9 region of the 18S rRNA gene.

	Parasites	Number of positive fecal samples (%)	Average reads	Location	Animal ID
Helminths	*Strongyloides* sp. 1	2 (8.71 %)	1.39	Hoengseong	S042, S043
	*Oscheius* sp.	1 (3.57 %)	3.29	Hoengseong	S028
Protozoa	*Entamoeba* sp.	16 (57.14 %)	758.18	Hoengseong	S024, S025, S026, S027, S029, S032, S033, S034, S036, S037, S041, S032, S046, S047, S048, S050
	*Sappinia* sp.	1 (3.57 %)	1.21	Hoengseong	S043
	*Eimeria* sp.	1 (3.57 %)	0.96	Hoengseong	S027

In Supplementary Table 4, the DNA sequences of all the identified parasites are provided. We discovered that *Strongyloides* sp. from the wild boars and water deer shared an identical sequence (*Strongyloides* sp. 1), whereas *Strongyloides* sp. from raccoon dogs and leopard cats shared an identical sequence (*Strongyloides* sp. 2), which differed from the aforementioned sequence of *Strongyloides* sp. 1 by 8 base pairs. Additionally, the sequences of *Ancylostoma* sp. and *Entamoeba* sp. were found to be consistent across all the examined hosts.

## 4 Discussion

In the present study, we detected *Metastrongylus* sp. in wild boars, which can cause severe lung pathologies, resulting in coughing and weight loss in infected animals [23]. Previously, *Metastrongylus elongatus* was detected in Korean wild boars [24] and *Metastrongylus* spp. infections were also reported in domestic pigs [25]. Furthermore, *Strongyloides* sp. 1 was detected in the wild boars in our study. *Strongyloides ransomi* has been observed in wild boars in Japan and pigs in Korea [26,27]. The *Strongyloides* DNA sequence detected in wild boars in our study could potentially be that of *Strongyloides ransomi*. In addition, infections with the protozoan *Entamoeba* sp. and *Blastocystis* sp. were also detected in wild boars in our study, which is consistent with findings from previous reports [28,29]. Furthermore, in a previous study, *Pentatrichomonas* sp. has been identified in the intestines of sheep and goats [30].

*Strongyloides* sp. 2 and *Ancylostoma* sp. were detected with a relatively higher prevalence in raccoon dogs than in the other animals investigated in this study (Table 2). In previous studies conducted in Korea, *Arthrostoma miyazakiense*, but not *Ancylostoma* sp., was found [31]. In our study, we used the NCBI database, where the 18S rRNA gene sequence of *Arthrostoma* sp. has not yet been registered. *Strongyloides planiceps* has been previously reported in raccoon dogs in Japan [32]. We also detected *Toxocara* sp. (40.34 %) in raccoon dogs, similar to previous findings of studies conducted in Korea and Japan [31,33]. In addition, in a previous study, *Toxocara tanuki* was detected in raccoon dogs in Korea [34]. *Panagrellus* sp. is considered a free-living nematode found in soil; it may be accidently ingested by raccoon dogs [35]. Two trematodes, Echinostomatidae gen. sp. and *Alaria* sp. were detected in our study. Previously, *Echinochasmus perfoliatus* and *Echinochasmus japonicus* were observed in Korean raccoon dogs [36,37]. Moreover, *Alaria alata* infection was recently confirmed in Korean raccoon dogs [38].

*Strongyloides* sp. 2 was detected in leopard cats in our study, which correlates with previous reports [[39], [40], [41]]. *Ancylostoma* sp. was also observed in the fecal sample of one leopard cat in our study. Previous studies have reported *Ancylostoma ceylanicum* infections in leopard cats [42]. However, in the present study, for the first time, we observed Echinostomatidae gen. sp. infection in Korean leopard cats. A previous report indicated that *Echinochasmus japonicus*, *Echinostoma revolutum*, and *Echinostoma hortense* are often observed in stray cats in Korea [43]. Since the DNA sequences of the 18S V9 region of *Echinostoma* sp. and *Echinochasmus* sp. are identical (100 %), further methods for distinguishing them are necessary (Supplementary Table S4). *Spirometra* sp. (40 %) and *Clonorchis* sp. (20.00 %) were detected only in leopard cats in our study (Table 3), and have been recorded in leopard cats in previous reports [39,44,45]. The detection of *Spirometra* and *Clonorchis* in leopard cats is notable because they are capable of carrying and transmitting zoonotic organisms. In this study, to the best of our knowledge, for the first time, we discovered the DNA sequence of *Neodiplostomum* sp. in leopard cats. This DNA sequence was identified as being 100 % identical to that of *Neodiplostomum* sp. in our analysis pipeline. However, it could also belong to another parasite sharing the same 18S rDNA sequence. In addition, it is possible that the sequence was accidently obtained during the digestion process after a leopard cat consumed another animal, as *Neodiplostomum* sp. is known to be a parasite of birds and reptiles. Indeed, in our study, avian 18S rDNA was also identified in the leopard cat stool sample that tested positive for *Neodiplostomum* sp. (data not shown). However, *Pharyngostomum cordatum* is more commonly detected in feral cats than *Neodiplostomum* sp. [41,46]. *Tritrichomonas* sp. and *Cryptosporidium* sp. were also observed in the fecal samples of leopard cats (Table 3). In previous studies, these protozoa have been found in cats but not in leopard cats [[47], [48], [49]].

In the present study, fewer parasitic infections were detected in water deer than in the other animals examined. *Strongyloides* sp. 1 was detected in water deer, similar to a previous report [50]. *Oscheius* sp. has been considered a free-living nematode that is sometimes found in soil, while other species are parasites of insects or slugs [51,52]. It is believed that they might be accidently ingested by water deer while grazing. *Entamoeba* sp., a protozoan, accounted for the majority of protozoa infections in water deer (57.14 %). To the best of our knowledge, our study is the first to report the presence of *Entamoeba* sp. and *Sappinia* sp. in water deer. *Sappinia* sp. is generally considered a free-living amoeba, but cases of amoebic encephalitis attributed to *Sappinia diploidea* have been previously reported [53,54]. Furthermore, *Eimeria* sp. was observed in the water deer, which aligns with a previous report [50].

Although the current study is comprehensive, we acknowledge that it has a few limitations. Firstly, the direct collection of environmental feces could introduce potential contamination; besides the contamination occurring during collection of fecal material, foreign parasitic DNA in the food material could be detected in the host feces. Secondly, we did not collect or identify parasitic worms or their eggs microscopically. Future studies that combine traditional methods, such as microscopic investigation, would be necessary to validate the findings of the current study. Finally, as we utilized the Illumina iSeq 100 system, which is known for its short sequence length coverage, there's an inherent limitation with regard to achieving the precise identification of parasite species. For example, in our study, although the DNA sequence of the 18S V9 region in *Strongyloides* sp. 1 detected in water deer is identical to that found in wild boars, there is a possibility that these hosts harbor different *Strongyloides* species [26,27,50]. Indeed, Table S4 indicates that *Strongyloides* sp. 1 is 100 % identical to *S. papillosus* in DNA sequence of 18S V9. This suggests that the 18S V9 region may not be a species-specific marker for identifying *Strongyloides* species, and further studies utilizing other regions of the 18S rDNA are necessary [55]. Similarly, although an identical DNA sequence of *Ancylostoma* sp. was detected in both leopard cats and raccoon dogs in our study, past literature indicates that they are probably different species [31,42,45,[56], [57], [58]]. Also, Table S4 indicates that the DNA sequence of *Ancylostoma* sp. found in this study is identical to those of multiple species such as *A. ceylanicum*, *A. caninum*, and even *Oswaldocruzia* sp. (Trichostrongyloidea). This indicates the necessity of the discovery of more effective target genes beyond the 18S V9 region to enhance the elucidation of parasite diversity in wild mammals.

An integrated approach involving wildlife management, veterinary science, public health, and environmental sectors remains crucial for addressing such parasitic risks. Furthermore, refining techniques and methodologies, especially aimed at eliminating potential contaminants, could improve the robustness of future studies. Beyond laboratory analysis, field surveys could provide nuanced perspectives into the ecology of the parasitic infections.

## 5 Conclusion

The present study analyzed fecal samples from four dominant species of wild animals to identify parasites by metabarcoding the 18S rRNA gene. Genes from various helminths and protozoa, such as *Metastrongylus* sp., *Strongyloides* spp., *Ancylostoma* sp., *Toxocara* sp., *Spirometra* sp., *Alaria* sp., *Clonorchis* sp., *Entamoeba* sp., *Blastocystis* sp., and *Cryptosporidium* sp., were detected. Using advanced surveillance techniques, such as metabarcoding of the 18S rRNA gene, offers a promising avenue for the efficient and effective control of pathogen transmission from wildlife to humans and other animals in the future.

## Data availability statement

Raw sequence data are available in NCBI GenBank under BioProject PRJNA932541. All data generated or analyzed during this study are included in this published article and its supplementary information files.

## CRediT authorship contribution statement

**Jun Ho Choi:** Writing – original draft. **Soo Lim Kim:** Investigation. **Dong Kyun Yoo:** Investigation. **Myung-hee Yi:** Formal analysis. **Singeun Oh:** Data curation. **Myungjun Kim:** Software. **Sohyeon Yun:** Methodology. **Tai-Soon Yong:** Conceptualization. **Seongjun Choe:** Data curation. **Jong Koo Lee:** Conceptualization. **Ju Yeong Kim:** Writing – review & editing, Supervision, Funding acquisition.

## Declaration of competing interest

The authors declare that they have no known competing financial interests or personal relationships that could have appeared to influence the work reported in this paper.
