# Supplementary material for: Metabarcoding of pathogenic parasites based on copro-DNA analysis of wild animals in South Korea
Source: Heliyon. 2024 Apr 25;10(9):e30059. doi: 10.1016/j.heliyon.2024.e30059 (PMC11066388; doi:10.1016/j.heliyon.2024.e30059)
Supplement: Multimedia component 1 [file mmc1.docx]

Supplementary Table S1. Information of the fecal samples

| No. | ID | species | Collection date | Collection city | GPS |
| --- | --- | --- | --- | --- | --- |
| 1 | S001 | *Sus scrofa coreanus* | November, 2021 | Hoengseong | 37.424805, 128.156821 |
| 2 | S002 | *Sus scrofa coreanus* | November, 2021 | Hoengseong | 37.424805, 128.156821 |
| 3 | S003 | *Sus scrofa coreanus* | November, 2021 | Hoengseong | 37.424805, 128.156821 |
| 4 | S024 | *Hydropotes inermis argyropus* | November, 2021 | Hoengseong | 37.447319, 128.112157 |
| 5 | S025 | *Hydropotes inermis argyropus* | November, 2021 | Hoengseong | 37.447319, 128.112157 |
| 6 | S006 | *Nyctereutes procyonoides koreensis* | November, 2021 | Hoengseong | 37.352177, 128.153534 |
| 7 | S007 | *Nyctereutes procyonoides koreensis* | November, 2021 | Hoengseong | 37.352177, 128.153534 |
| 8 | S008 | *Nyctereutes procyonoides koreensis* | December, 2021 | Hoengseong | 37.402467, 128.148166 |
| 9 | S009 | *Nyctereutes procyonoides koreensis* | December, 2021 | Hoengseong | 37.403175, 128.148269 |
| 10 | S010 | *Nyctereutes procyonoides koreensis* | December, 2021 | Hoengseong | 37.404276, 128.149914 |
| 11 | S011 | *Nyctereutes procyonoides koreensis* | December, 2021 | Hoengseong | 37.404155, 128.15014 |
| 12 | S012 | *Nyctereutes procyonoides koreensis* | December, 2021 | Hoengseong | 37.404153, 128.15024 |
| 13 | S013 | *Nyctereutes procyonoides koreensis* | December, 2021 | Hoengseong | 37.403144, 128.150483 |
| 14 | S014 | *Nyctereutes procyonoides koreensis* | December, 2021 | Hoengseong | 37.402639, 128.150994 |
| 15 | S019 | *Prionailurus bengalensis euptilurus* | December, 2021 | Hoengseong | 37.352801, 128.156291 |
| 16 | S020 | *Prionailurus bengalensis euptilurus* | December, 2021 | Hoengseong | 37.352204, 128.153521 |
| 17 | S015 | *Nyctereutes procyonoides koreensis* | December, 2021 | Hoengseong | 37.352177, 128.153534 |
| 18 | S016 | *Nyctereutes procyonoides koreensis* | December, 2021 | Hoengseong | 37.35219, 128.153402 |
| 19 | S026 | *Hydropotes inermis argyropus* | December, 2021 | Hoengseong | 37.422756, 128.158371 |
| 20 | S027 | *Hydropotes inermis argyropus* | December, 2021 | Hoengseong | 37.422808, 128.157914 |
| 21 | S028 | *Hydropotes inermis argyropus* | December, 2021 | Hoengseong | 37.423441, 128.157607 |
| 22 | S029 | *Hydropotes inermis argyropus* | December, 2021 | Hoengseong | 37.424805, 128.156821 |
| 23 | S030 | *Hydropotes inermis argyropus* | December, 2021 | Hoengseong | 37.424722, 128.156579 |
| 24 | S031 | *Hydropotes inermis argyropus* | December, 2021 | Hoengseong | 37.4247, 128.156556 |
| 25 | S032 | *Hydropotes inermis argyropus* | December, 2021 | Hoengseong | 37.442215, 128.060569 |
| 26 | S033 | *Hydropotes inermis argyropus* | December, 2021 | Hoengseong | 37.443268, 128.060037 |
| 27 | S034 | *Hydropotes inermis argyropus* | December, 2021 | Hoengseong | 37.44307, 128.060358 |
| 28 | S035 | *Hydropotes inermis argyropus* | December, 2021 | Hoengseong | 37.46323, 128.058458 |
| 29 | S036 | *Hydropotes inermis argyropus* | December, 2021 | Hoengseong | 37.461806, 128.05994 |
| 30 | S037 | *Hydropotes inermis argyropus* | December, 2021 | Hoengseong | 37.461817, 128.060223 |
| 31 | S038 | *Hydropotes inermis argyropus* | December, 2021 | Hoengseong | 37.461951, 128.061033 |
| 32 | S039 | *Hydropotes inermis argyropus* | December, 2021 | Hoengseong | 37.462244, 128.061285 |
| 33 | S040 | *Hydropotes inermis argyropus* | December, 2021 | Hoengseong | 37.461198, 128.060043 |
| 34 | S041 | *Hydropotes inermis argyropus* | December, 2021 | Hoengseong | 37.324695, 128.072851 |
| 35 | S042 | *Hydropotes inermis argyropus* | December, 2021 | Hoengseong | 37.324341, 128.072442 |
| 36 | S043 | *Hydropotes inermis argyropus* | December, 2021 | Hoengseong | 37.324272, 128.071862 |
| 37 | S044 | *Hydropotes inermis argyropus* | December, 2021 | Hoengseong | 37.442847, 128.060709 |
| 38 | S045 | *Hydropotes inermis argyropus* | December, 2021 | Hoengseong | 37.442043, 128.060835 |
| 39 | S046 | *Hydropotes inermis argyropus* | December, 2021 | Hoengseong | 37.442215, 128.060569 |
| 40 | S047 | *Hydropotes inermis argyropus* | December, 2021 | Hoengseong | 37.324244, 128.082613 |
| 41 | S048 | *Hydropotes inermis argyropus* | December, 2021 | Hoengseong | 37.323977, 128.081799 |
| 42 | S049 | *Hydropotes inermis argyropus* | December, 2021 | Hoengseong | 37.323983, 128.080008 |
| 43 | S050 | *Hydropotes inermis argyropus* | December, 2021 | Hoengseong | 37.341935, 128.098186 |
| 44 | S051 | *Hydropotes inermis argyropus* | December, 2021 | Hoengseong | 37.340757, 128.100478 |
| 45 | S021 | *Prionailurus bengalensis euptilurus* | January, 2022 | Cheongyang | 36.4134003, 126.88428 |
| 46 | S022 | *Prionailurus bengalensis euptilurus* | January, 2022 | Cheongyang | 36.4134003, 126.88428 |
| 47 | S023 | *Prionailurus bengalensis euptilurus* | January, 2022 | Yangpyeong | 37.544325, 127.567490 |
| 48 | S017 | *Nyctereutes procyonoides koreensis* | January, 2022 | Cheongyang | 36.4303883, 126.8089009 |
| 49 | S018 | *Nyctereutes procyonoides koreensis* | January, 2022 | Cheongyang | 36.4303883, 126.8089009 |
| 50 | S004 | *Sus scrofa coreanus* | January, 2022 | Hoengseong | 37.44815, 128.112562 |
| 51 | S005 | *Sus scrofa coreanus* | January, 2022 | Hoengseong | 37.448167, 128.111855 |
