# Supplementary material for: Metabarcoding of pathogenic parasites based on copro-DNA analysis of wild animals in South Korea
Source: Heliyon. 2024 Apr 25;10(9):e30059. doi: 10.1016/j.heliyon.2024.e30059 (PMC11066388; doi:10.1016/j.heliyon.2024.e30059)
Supplement: Multimedia component 2 [file mmc2.docx]

Supplementary Table S2. Read count per sample after metabarcoding of 18S and 12S rRNA gene amplicon analysis.

|  | sample-frequency | |
| --- | --- | --- |
| ID | 18S | 12S |
| S001 | 62391 | 35741 |
| S002 | 53372 | 18886 |
| S003 | 58357 | 15888 |
| S024 | 5543 | 13819 |
| S025 | 54833 | 13458 |
| S006 | 71479 | 51393 |
| S007 | 63444 | 3923 |
| S008 | 59283 | 19002 |
| S009 | 35920 | 28359 |
| S010 | 39007 | 34993 |
| S011 | 22756 | 8249 |
| S012 | 16683 | 30261 |
| S013 | 26079 | 7607 |
| S014 | 22270 | 3196 |
| S019 | 61074 | 4340 |
| S020 | 76219 | 3290 |
| S015 | 80325 | 70246 |
| S016 | 79179 | 45702 |
| S026 | 89694 | 24452 |
| S027 | 49241 | 32893 |
| S028 | 61584 | 34864 |
| S029 | 49523 | 5250 |
| S030 | 57048 | 31483 |
| S031 | 55448 | 33067 |
| S032 | 59862 | 2306 |
| S033 | 73036 | 42187 |
| S034 | 73808 | 21633 |
| S035 | 23410 | 14392 |
| S036 | 79523 | 29493 |
| S037 | 70251 | 24236 |
| S038 | 85101 | 53337 |
| S039 | 86235 | 65925 |
| S040 | 89137 | 1581 |
| S041 | 83322 | 41389 |
| S042 | 82102 | 60774 |
| S043 | 83443 | 65227 |
| S044 | 81116 | 36281 |
| S045 | 67698 | 46411 |
| S046 | 67468 | 58418 |
| S047 | 24535 | 48669 |
| S048 | 68368 | 81925 |
| S049 | 70322 | 58874 |
| S050 | 23238 | 47679 |
| S051 | 58777 | 35045 |
| S021 | 67242 | 44279 |
| S022 | 65622 | 4441 |
| S023 | 67585 | 33429 |
| S017 | 71000 | 50313 |
| S018 | 75281 | 46623 |
| S004 | 69449 | 45398 |
| S005 | 81146 | 11817 |
